# Non‐steroidal anti‐inflammatory drugs increase urinary neutrophil gelatinase‐associated lipocalin in recreational runners

**DOI:** 10.1111/sms.13755

**Published:** 2020-08-13

**Authors:** Khrystyna O. Semen, Rick H. A. van der Doelen, Monique van der Lugt, Davy G. H. A. van Dam, Jürgen Reimer, Frank R. M. Stassen, Loes Janssen, Paddy K. C. Janssen, Marcel J. W. Janssen, Aalt Bast, Jos L. M. L le Noble

**Affiliations:** ^1^ Campus Venlo Maastricht University Venlo The Netherlands; ^2^ Department of Clinical Chemistry VieCuri Medical Center Noord‐Limburg Venlo The Netherlands; ^3^ Emergency Department VieCuri Medical Center Noord‐Limburg Venlo The Netherlands; ^4^ Department of Internal Medicine VieCuri Medical Center Noord‐Limburg Venlo The Netherlands; ^5^ Medcaptain Europe BV Andelst The Netherlands; ^6^ Department of Medical Microbiology NUTRIM – School of Nutrition and Translational Research in Metabolism Maastricht University Maastricht The Netherlands; ^7^ Department of Epidemiology VieCuri Medical Center Noord‐Limburg Venlo The Netherlands; ^8^ Department of Clinical Pharmacy VieCuri Medical Center Noord‐Limburg Venlo The Netherlands; ^9^ Department of Clinical Pharmacy and Toxicology Maastricht University Medical Center Maastricht The Netherlands; ^10^ Department of Pharmacology and Toxicology Maastricht University Maastricht The Netherlands; ^11^ Department of Intensive Care VieCuri Medical Center Noord‐Limburg Venlo The Netherlands

**Keywords:** half‐marathon, NSAIDs, renal function, running, urinary neutrophil gelatinase‐associated lipocalin (uNGAL)

## Abstract

**Objectives:**

To study the effects of running with/without the use of pain killers on urinary neutrophil gelatinase‐associated lipocalin (uNGAL) and other parameters of kidney function in recreational runners.

**Methods:**

Participants of the 10‐ and 21.1‐km Weir Venloop race were enrolled and their urine samples collected before and after the run. Urine dipstick and other conventional tests used to assess kidney function were performed. The presence of ibuprofen, diclofenac, naproxen, and/or paracetamol was assessed by LC‐MS/MS. uNGAL was measured with a two‐step chemiluminescent immunoassay.

**Results:**

NSAIDs/analgesics were detected in urine of 5 (14.4%) 10‐km runners and 13 (28.9%) 21.1‐km runners. Only half‐marathon participants showed significant increases in uNGAL (pre: 11.7 [7.1‐34.3] ng/mL; post: 33.4 [17.4‐50.4] ng/mL; *P* = .0038). There was a significant effect of NSAID/analgesic use on uNGAL increase (F_2, 76_ = 4.210, *P* = .004). Post hoc tests revealed that uNGAL increased significantly in runners who tested positive for ibuprofen/naproxen compared to runners who did not use any medications (*P* = .045) or those who tested positive for paracetamol (*P* = .033). Running distance had a significant influence on the increase in uNGAL (F_1, 53_ = 4.741, *P* < .05), specific gravity (F_1, 60_ = 9.231, *P* < .01), urinary creatinine (F_1, 61_ = 10.574, *P* < .01), albumin (F_1, 59_ = 4.888, *P* < .05), and development of hematuria (χ^2^(4) = 18.44, *P* = .001).

**Conclusions:**

Running distance and use of ibuprofen/naproxen were identified as risk factors for uNGAL increase in recreational runners.

## 1 INTRODUCTION

Long‐distance running is an increasingly practiced form of endurance exercise.^1^ The popularity of this sport is attributed to its multiple potential health benefits and relatively easy accessibility. Runners, in addition to having a lower risk of diabetes and cancer, demonstrate a 25%‐40% reduced risk of premature mortality and live approximately 3 years longer than non‐runners.^2^, ^3^, ^4^ Distances of 21.1 km and below are frequently chosen by recreational athletes due to their challenging but less physically and mentally demanding nature compared with participation in a full marathon (42.2 km).^5^

Changes in physiological functions during long‐distance running were shown to promote the development of various adverse events involving the kidney.^6^ Redistribution of the cardiac output during exercise reduces renal perfusion up to 25% compared to the rest levels, which may lead to ischemic damage and transient decrease in the renal function.^7^ Although such a response is commonly regarded as a physiological reaction to exercise stress, some cases progress to clinically significant acute kidney injury (AKI) or even acute kidney failure.^8^, ^9^, ^10^ The prevalence of AKI in runners has been traditionally assessed by the measurement of serum creatinine (SCr).^8^, ^10^, ^11^, ^12^ The major criticism of this biomarker is attributed to the fact that it primarily reflects filtration processes and does not give information about tubular damage. Moreover, SCr tends to peak rather late (48‐72 hours) after insult which makes it unsuitable for an early detection of AKI and identification of at‐risk subjects.^13^ Several novel biomarkers of kidney injury have been proposed,^9^, ^11^ with urinary neutrophil gelatinase‐associated lipocalin (uNGAL) reported to be reliable for the assessment of renal function in long‐distance runners.^14^, ^15^

Intake of non‐steroidal anti‐inflammatory drugs (NSAIDs) was shown to increase the risk of adverse effects in recreational runners.^16^ NSAIDs further decrease the rate of glomerular filtration and compromise renal blood supply during exercise via inhibition of the production of the vasodilatory prostaglandins (PGI_2_) in the afferent arterioles of the kidney. Use of NSAIDs before/during the race was reported in 45.9%‐49% of the marathoners^17^, ^18^ and in 60.3%‐70% of the ultramarathon runners.^19^, ^20^ Among the half‐marathon runners, the intake of NSAIDs/analgesics was reported in 8.3% (CI 95%: 8.1‐8.6) of the race entrants.^21^ These studies involved recall questionnaires with various recall windows and presence of medication in participant biological fluids was not confirmed. Screening for NSAIDs/analgesics in urine during the competition may provide more accurate information about their use by recreational runners and could help to eliminate possible recall bias related to low recognition of risks associated with those medications.

This study aimed to evaluate the prevalence of NSAIDs/analgesics use by urine testing in recreational 10‐ and 21.1‐km runners. Furthermore, it intended to establish the effects of running with/without the use of NSAIDs/analgesics on the levels of urinary neutrophil gelatinase‐associated lipocalin (uNGAL) and conventional biomarkers of kidney function.

## 2 MATERIALS & METHODS

### 2.1 Participants

The study participants were enrolled from among the runners registered for the Weir Venloop contest. Email invitations were sent to the contestants by the competition organizers. Contestants aged ≥18 years, with at least 2 years of running experience and at least two races (>10 km in distance) completed in the last 3 years, were included in this study. A history of major running injuries in the 3 months prior to the competition and a presence of chronic kidney failure were defined as the exclusion criteria. The study was conducted in accordance with the Declaration of Helsinki on biomedical research involving human subjects. Approval was obtained from the institutional review board of the VieCuri Medical Centre (Venlo, the Netherlands). Prior to inclusion into this study, all participants signed the informed consent.

### 2.2 Design and procedures

This was a single‐blind cohort study. The runners who consented to participate were asked to fill in a questionnaire about their demographics, training history, medical history, and use of medications over the month before the race. Body weight was measured before and after the race, and BMI was calculated. Urine samples were collected twice: 30‐90 minutes before the race (start sample) and shortly after the finish (finish sample). Immediately upon collection, a fresh portion of the urine was taken for analysis with urine dipsticks. The rest of the urine was stored at 4‐7°C and protected from direct sunlight until the last participant delivered the finish sample. Subsequently, all samples were transported to the Laboratory of Clinical Chemistry and Hematology of the VieCuri Medical Centre, where they were aliquoted and stored at −80°C for further analysis. The finishing times of the participants were obtained from the Weir Venloop website (http://www.venloop.nl).

### 2.3 Laboratory measurements

Specific gravity was measured by the refractometer (UN‐4000 analyzer, Sysmex Corporation, Kobe, Japan). Albumin, creatinine, and sodium were assessed by bromocresol purple, enzymatic (creatininase/creatinase/sarcosine oxidase), and indirect ion‐selective electrode assays, respectively, using the Architect c8000 analyzer (Abbott Diagnostics, Abbott Park, IL, USA) with proprietary reagents. NGAL was measured with a two‐step chemiluminescent immunoassay using Architect i2000 analyzer (Abbott Diagnostics).^22^ Analyses with urine dipsticks were performed using meditape UC‐10S (Sysmex) urinalysis test strips.

Both pre‐ and post‐race urine samples were tested for NSAIDs/analgesics. Prior to measurement, the urine samples were treated with beta‐glucuronidase, after which paracetamol, ibuprofen, diclofenac, and naproxen were quantified by an in‐house validated LC‐MS/MS method using Acquity UPLC H‐class system and Xevo TQD mass spectrometer (Waters Corporation). A case was considered positive for the intake of NSAIDs/analgesics when at least one of two samples was found to be positive for the tested compounds.

### 2.4 Statistics

All statistical tests have been carried out using SPSS (version 22, IBM Corporation). Only data from the participants with start and finish urine sample available were analyzed. The results for the categorical variables are presented as percentage and for numerical data as the mean ± SEM or as median [interquartile range]. Paired Student's *t* tests (two‐tailed) were used to test parametric data, Wilcoxon matched‐pairs signed‐rank test was used for non‐parametric data, and chi‐square test was used for the categorical data. Factorial ANOVA was applied to reveal effects of NSAIDS/analgesics and distance on the changes in uNGAL and other parameters of renal function. For this purpose, the “delta” levels were calculated by subtracting the start levels from the finish levels for each urinary parameter. For NSAID/analgesics, participants were subdivided into three categories: paracetamol, NSAID (ibuprofen/diclofenac/naproxen), or no detection. Age, BMI, and training were applied as covariates in the factorial ANOVA for those delta measures that showed a significant correlation in the Spearman analysis (*P* < .05). The delta urinary analytes were considered eligible for ANOVA after a visual assessment of their distribution histograms. If the factorial ANOVA revealed significant effects of NSAID/analgesics or distance, the sample was accordingly split for one‐way ANOVA with Bonferroni post‐hoc testing for NSAID/analgesics or the Wilcoxon signed‐rank test for distance. The three‐way log linear analysis was used to assess the interaction between the distance, use of NSAIDs/analgesics, and changes in the urine dipstick. The differences were considered statistically significant when *P* < .05.

## 3 RESULTS

### 3.1 Participants

**Figure 1:**
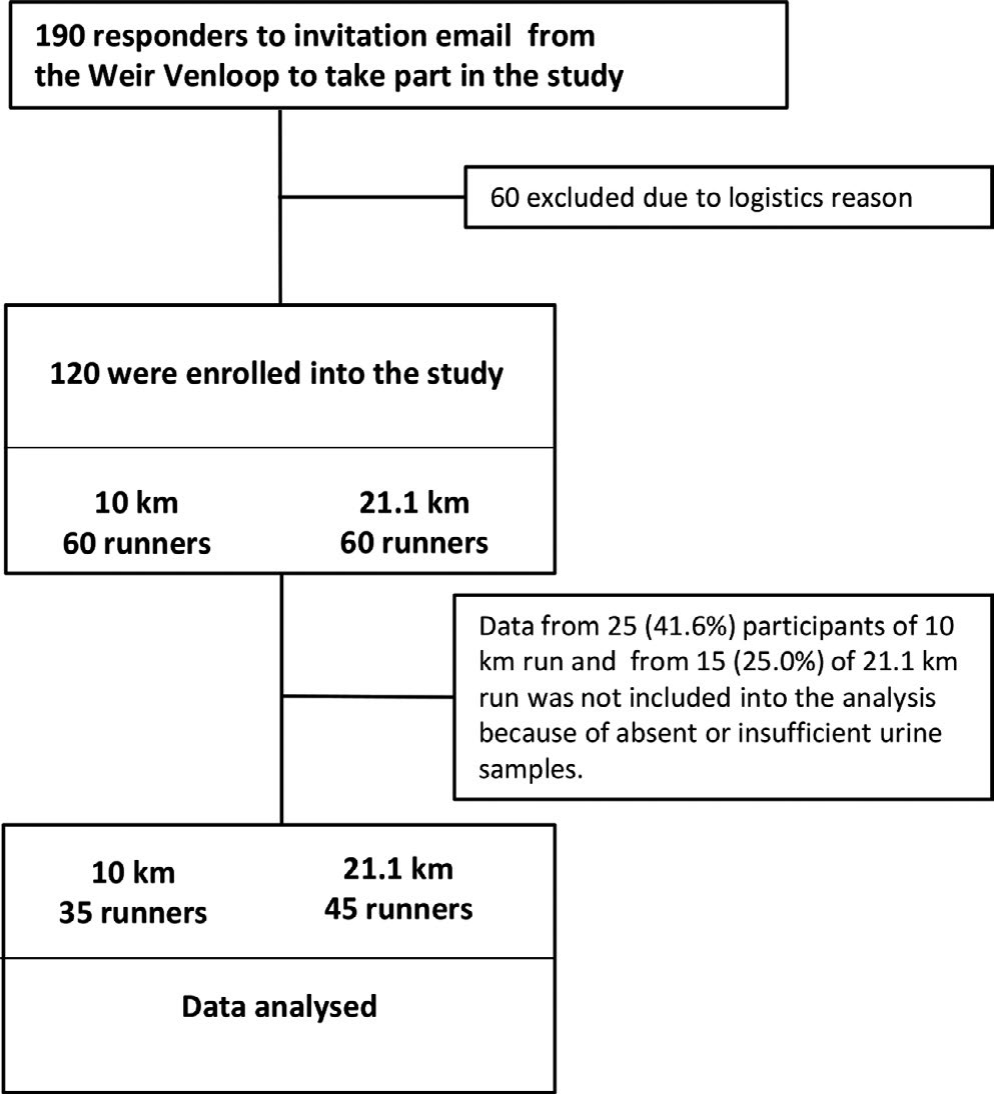
Enrollment chart of runners into the study cohort

For logistic reasons, only 120 participants (60 for 10‐km and 60 for 21.1‐km distance) out of the 190 responders of the online survey who met the inclusion criteria were included in the study (Figure 1). On the day of the Weir Venloop contest, the weather was cloudy with no rain, the temperature ranged between 4 and 7°C, and the average humidity was 77%. The mean age of the study participants was 49 ± 13 years, and 59 (49.2%) runners were male. Both start and finish urine samples were available for 35 runners (44 ± 2 years, 51.4% male) of the 10‐km run and for 45 (55 ± 2 years, 46.7% male) half‐marathoners. Pre‐race samples were not collected from three of the 10‐km runners and by eight half‐marathon contestants. Post‐race urine was not obtained for 25 runners of the 10‐km distance and for 12 persons participating in half‐marathon. The reasons for missing samples included an inability to urinate within 90 minutes after the run and poor compliance.

Characteristics of the runners who provided urine both before and after the race are summarized in Table 1, and data from only these individuals were further analyzed. The male participants of the half‐marathon were significantly older and had higher BMI compared to the female participants (*P* < .01), and the 10‐km participants were significantly younger than the 21.1‐km participants (*P* < .001). No major differences in training load between women and men within the particular running distance group were noted. Use of medication over the one month before the competition was reported by 13 (37.5%) runners of the 10‐km race and 17 (37.8%) runners of half‐marathon. Interestingly, the use of NSAIDs/analgesics tested in this study was noted only by one athlete participating in the half‐marathon (Supplementary Table 1). The weight lost after running 10 km was 1.1 [0.3‐2.0] kg and after running 21.1 km was 1.8 [1.0‐2.9] kg.

**Table 1:** Characteristics and post‐running outcomes of the study participants

Characteristics	10 km (n = 35)	21.1 km (n = 45)
Male/female	18/17	21/24
Age, y		
Male	47 ± 4	62 ± 1* #
Female	40 ± 2	48 ± 2
BMI, kg/m^2^		
Male	24.8 ± 0.5*	24.4 ± 0.4*
Female	22.6 ± 0.4	22.4 ± 0.4
Training, km/week		
Male	15 (4‐20)	34 (10‐50)
Female	17 (7‐50)	29 (10‐40)
Post‐running outcomes		
Weight loss, kg	1.1 [0.3‐2.0]	1.8 [1.0‐2.9]
NSAIDs/analgesics detected in urine (%)		
Paracetamol	‐	8 (17.8)
Ibuprofen	3 (8.6)	4 (8.9)
Diclofenac	‐	‐
Naproxen	2 (5.7)	‐
Paracetamol and ibuprofen	‐	1 (2.2)
Total	5 (14.4)	13 (28.9)

Note

Categorical values are presented as number (percentage); values for continuous variables are presented as median [range] or mean ± SEM.

Abbreviations: AKI, acute kidney injury; BMI, body mass index; NSAIDs, non‐steroidal anti‐inflammatory drugs.

* Difference significant between male and female within 10 or 21.1 km run.

^#^ Significant difference between 10 and 21.1 km.

### 3.2 Use of NSAIDs/analgesics

Five (14.4%) of 10‐km runners and 13 (28.9%) of the half‐marathoners showed the presence of NSAIDs/analgesics in the urine samples (Table 1). Paracetamol was detected in eight (17.8%) of the 21.1‐km participants and in none of the 10‐km runners. Naproxen was found in the urine of two (5.7%) 10‐km runners, and the presence of ibuprofen was confirmed in three (8.6%) and four (8.9%) runners of 10 km and 21.1 km, respectively. Urine of one half‐marathon runner was positive for both paracetamol and ibuprofen. No evidence of diclofenac use by the study participants was documented.

### 3.3 Changes in uNGAL

**Figure 2:**
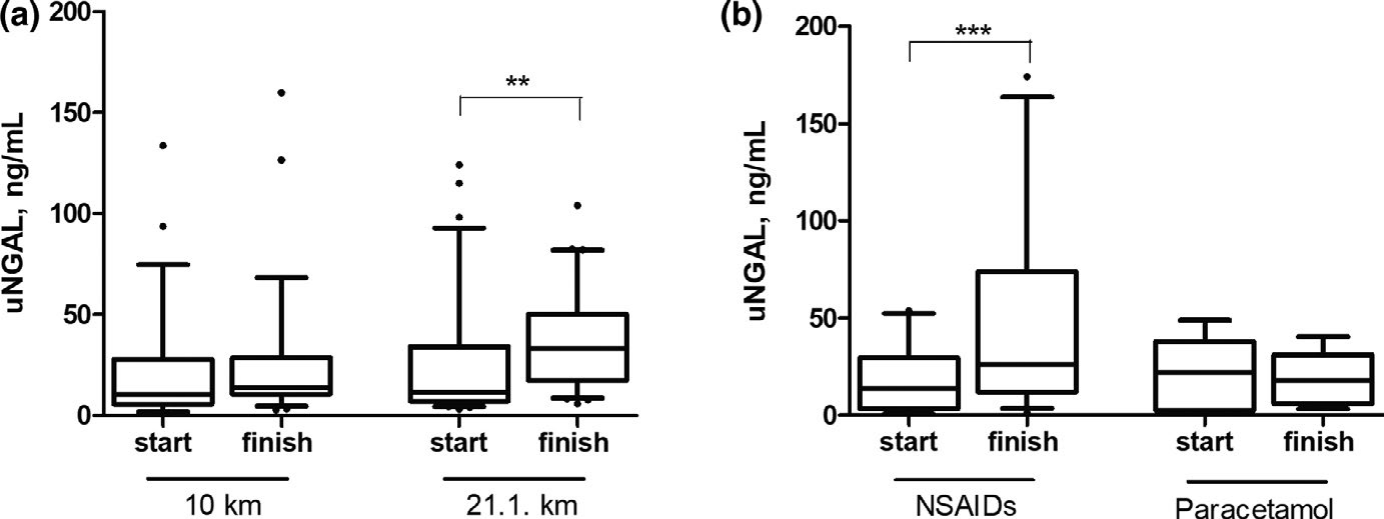
Changes in urinary neutrophil‐associated lipocalin (uNGAL) with running alone (A) and with concomitant use of NSAIDs/analgesics (B). NSAID stands for ibuprofen and/or naproxen. Significance was assessed by the Wilcoxon matched‐pairs signed‐rank test (data presented as box, whiskers 10‐90 percentile)

Running 21.1 km caused a significant increase in uNGAL (Figure 2). Interestingly, this increase was not observed in the 21.1‐km runners who tested positive for paracetamol (mean difference 11.95 [95% CI: −15.2; 39.8] ng/mL, *P* = .853) and was further potentiated by the intake of ibuprofen/naproxen (mean difference 40.2 [95% CI: 6.1; 74.3] ng/mL, *P* = .022). Completing 10‐km run caused an increase in uNGAL only when combined with ibuprofen/naproxen use (*P* = .031; Figure 2).

There was a significant effect of NSAID/analgesic use on uNGAL (F_2,53_ = 5.335, *P* < .01). The Bonferroni post‐hoc test revealed that the levels of uNGAL were significantly higher in runners who tested positive for ibuprofen/naproxen than in runners who did not use any medication (*P* = .045) or those who tested positive for paracetamol (*P* = .033). Running distance had a significant influence on the increase of uNGAL (F_1,53_ = 4.741, *P* < .05).

### 3.4 Changes in the conventional parameters characterizing kidney function

Exercise‐induced stress on the kidneys was noted from changes in the conventional parameters characterizing renal function. Runners completing either a 10‐ or 21.1 km race had increased levels of urinary albumin and albumin/creatinine ratio (Table 2). Urinary creatinine and specific gravity increased only in the 21.1‐km runners but not in 10‐km runners. No major changes of urinary sodium, uric acid, glucose levels, and ketones (data not shown) were observed with running. There was no effect of NSAIDs/analgesics on the levels of urinary creatinine, albumin, albumin/creatinine ratio, uric acid, and sodium. At the same time, the running distance was significantly related to the changes in urine‐specific gravity (F_1, 60_ = 9.231, *P* < .01), urinary creatinine (F_1, 61_ = 10.574, *P* < .01), and albumin (F_1, 59_ = 4.888, *P* < .05) observed with running. Urine dipstick testing revealed more cases of post‐race hematuria in the 10‐km runners (31 [88.6%] 10‐km runners vs. 25 [55.6%] half‐marathoners, χ^2^ (4) = 18.44, *P* = .001, Table 2). The three‐way loglinear analysis demonstrated no significant interaction between running distance, use of NSAIDs/analgesics, and degree of hematuria after the run (χ^2^(4) = 1.74, *P* = .783). At the same time, the frequency of low grade hematuria was similar in the 10‐ and 21.1‐km runners, while high grade hematuria was observed only in five (11.5%) half‐marathon runners.

**Table 2:** Variation in the conventional parameters characterizing the kidney function before and after 10 and 21.1 km run

	10 km, n = 35		21.1 km, n = 45	
	Before	After	Before	After
Specific gravity, kg/L	1.014 ± 0.002	1.010 ± 0.002*	1.010 ± 0.001	1.015 ± 0.001**^#^
Creatinine, mmol/L	5.18 [1.93‐11.81]	4.31 [2.46‐8.87]	3.45 [1.66‐6.62]	7.15 [4.78‐11.22]***^#^
Albumin, mg/L	2.5 [2.5‐11.5]	11.4 [5.7‐35.2]**	2.5 [2.5‐5.4]	27.0 [10.9‐77.6]***^#^
Albumin/creatinine, U	1.03 [0.67‐2.23]	3.95 [2.03‐5.56]***	1.12 [0.65‐1.89]	4.09 [1.59‐1.89]***
Uric acid, mmol/L	1.40 [0.65‐2.97]	0.82 [0.48‐2.15]	1.04 [0.6‐2.07]	1.3 [0.85‐1.7]
Sodium, mmol/L	52 [29‐104]	44 [29‐69]	52 [30‐81]	47 [34‐65]
Hematuria, n (%)				
1+	1 (3.2)	13 (37.1)	4 (8.9)	15 (33.3)
2+	‐	15 (42.9)	‐	4 (8.9)
3+	‐	3 (8.6)	‐	1 (2.2)
4+	‐	‐	‐	5 (11.1)
Total	1 (3.2)	1 (3.2)	4 (8.9)	25 (55.6)^£^

Note

Data presented as mean ± SEM, median (IQR), or number (%). Difference within groups was tested with Wilcoxon marched‐pairs signed‐rank test or paired *t* test with ^*^ *P* < .05, ^**^ *P* < .01, ^***^ *P* < .001.

^a^ *P* < .05.

^b^ *P* < .01.

^c^ *P* < .001.

^#^ Difference significant by factorial ANOVA.

^£^ Presence of hematuria was compared with chi‐square test.

## 4 DISCUSSION

It is known that recreational long‐distance runners frequently use NSAIDs/analgesics before/during the competition in order to prevent exercise‐induced pain and improve performance.^18^, ^21^, ^23^ Self‐reported intake of those medications by the ultramarathon, marathon, and half‐marathon participants varied between 35% and 72%.^6^, ^18^, ^24^ Interestingly, a recent study involving 76,654 race entrants of the 21.1‐ and 56‐km runs showed that only 12.2% (CI 95%, 12.0‐12.5) of the respondents reported the use of NSAIDs/analgesics before/during the running event.^21^ Thus, the estimated prevalence of the NSAIDs/analgesics used in relationship with endurance running varies significantly among different studies and requires further investigation.

This study showed that urine samples of 32.5% half‐marathoners and 15.6% of the 10‐km contestants were positive for the presence of NSAIDs/analgesics. To our knowledge, this is the first report to objectively verify the use of pain killers by recreational runners.

In contrast to the findings of previous studies, we demonstrated that ibuprofen or diclofenac were most frequently used by runners.^18^, ^21^, ^23^ At the same time, a surprisingly high number of participants in the current study tested positive for paracetamol. Low self‐reporting levels of NSAIDs/analgesics used in relation to the running event in our study may be explained by a low perception of risks related to the occasional intake of these medications. This further validates the concerns expressed by other researchers about the lack of knowledge among runners about the potential side effects of pain killers.^18^, ^23^, ^25^

Another important finding of this study is related to the influence of running on the kidney. One of the best novel biomarkers proposed for the assessment of the kidney damage in runners is uNGAL.^11^, ^14^, ^26^ uNGAL is a disulfide‐bridged polypeptide with a molecular weight of 25 kDa.^27^ It is expressed in response to stressful conditions and is used to assess the presence of acute kidney injury and tubular dysfunction in various clinical settings.^14^, ^28^, ^29^ A variety of tissues and cells, including the kidney, neutrophils, intestines, airways, and epithelia, have been shown to produce NGAL and contribute to its levels if the serum.^14^, ^15^, ^30^ In the kidneys, NGAL is produced by tubular epithelium in the loop of Henle, as well as collecting ducts, as a response to injury. NGAL is predominately released in the urine and not in systemic circulation. Therefore, the levels of NGAL in the urine are regarded as a good estimate of tubular damage.^31^ Systemically produced NGAL (eg, by activated neutrophils during strenuous exercise) is taken up by the proximal renal tubule, but it may also appear in the urine with injury to the proximal tubule.^32^ Therefore, tubular injury is an essential pathogenetic event leading to increased uNGAL. We cannot completely exclude the role of exercise‐induced neutrophil‐associated tissue damage and oxidative stress, together with decreased renal blood flow, in exercise‐induced renal dysfunction.^33^, ^34^

The superiority of NGAL measurement in urine over serum in exercise settings was substantiated by the independence of uNGAL and serum creatinine dynamics and, thus, less dependence on non‐renal factors such as exercise‐related muscle damage.^14^ An increase of 29.4 mg/dL or 38.8 mg/dL in uNGAL has been demonstrated in marathon runners.^10^, ^11^ In ultra‐endurance events uNGAL increased from 4.4 mg/dL to 35.6 ng/mL after 60 km and from 17 mg/dL to 38 ng/mL after 100 km.^14^, ^35^ The use of various detection methods in those studies (eg, electroluminescence, ELISA) may be responsible for some variability in the results.

The present study showed that running a half‐marathon is associated with an increase in uNGAL. Use of NSAIDs (ibuprofen/naproxen), which is a frequent practice in recreational and professional athletes, further contributed to the elevation of uNGAL, confirming that the use of those medications may increase the risk of kidney damage in runners.^16^, ^18^ Interestingly, the intake of the weak cyclooxygenase 1/2 inhibitor paracetamol did not change the levels of uNGAL compared with ibuprofen/naproxen intake. In the previous study, which involved exposure of volunteers to stress conditions simulating endurance exercise, paracetamol did not influence the kidney function, while ibuprofen caused significant reduction in the glomerular filtration rate and urine production.^36^ It can be assumed that, in the settings of vigorous exercise, paracetamol may be a relatively safe option in situations when the use of a pain killer cannot be avoided.

Post‐race hematuria and proteinuria described in this study are well‐documented metabolic consequences of vigorous exercise. While hematuria can result either from foot‐strike hemolysis, glomerular/urinary tract injury, or exercise‐induced muscle injury, albuminuria results primarily from exercise‐induced damage to the kidney parenchyma, with increased glomerular permeability and impaired tubular reabsorption. Previously, it was suggested that the degree of exercise‐related proteinuria correlates with exercise intensity rather than with its duration.^7^ In our study, increases in urinary albumin levels tended to be more prominent in half‐marathon participants compared to athletes running 10 km, suggesting that longer duration may also play role in the consequent degree of proteinuria in athletes. It remains unclear why the 10‐km runners developed hematuria more frequently compared to the half‐marathoners. Higher levels of physical fitness and increased adaptation of kidneys to long‐distance running may be a plausible explanation for this effect.

The major limitations of this study included a relatively small sample size. Moreover, it remains unclear to which extent an increase in uNGAL can be predictive of AKI as defined by the measurement of conventional biomarker serum creatinine. In the population undergoing cardiac surgery, the cutoff values of uNGAL suggestive of AKI ranged from 17 to 75 ng/mL by the ARCHITECT assay (same as used in this study).^37^ Due to different mechanisms contributing to the development of kidney dysfunction in these studies (eg, bypass surgery‐related hypothermia and perioperative thermodilution rather than exercise‐induced hyperthermia, hypovolemia, rhabdomyolysis), it is difficult to determine to which extent those results can be extrapolated to the runner population. In our opinion, the increase in uNGAL may be interpreted as a mild renal stress by analogy to the exercise‐induced increase in cardiac troponin that has been interpreted as a sign of mild cardiac stress and reversible cardiac injury.^38^

## 5 PERSPECTIVE

Risks associated with the intake of NSAIDs before/during long‐distance running are well recognized by medical professionals but not always by athletes. Evidence from this questionnaire‐based research suggests that a relatively large proportion of recreational runners use painkillers before/during the competition in order to prevent exercise‐induced pain and improve performance. Our study showed a discrepancy between the reported use of NSAIDs/painkillers and the results of urine testing, which might be attributed to the unawareness among runners about the potential adverse effects of those over‐the‐counter medications. Additional efforts should be taken by coaches, sports physiologists, and public health services to increase knowledge about the risks related to NSAID use while running. Importantly, the intake of NSAIDs further contributed to the development of renal stress during running. In situations when the use of painkiller before/during long‐distance running cannot be avoided, paracetamol must be the preferred option in terms of its sparing effect on the kidney. However, its safety must be further investigated in clinical trials.

## Supporting information

Supplementary Material
