# Supplementary material for: Non‐steroidal anti‐inflammatory drugs increase urinary neutrophil gelatinase‐associated lipocalin in recreational runners
Source: Scand J Med Sci Sports. 2020 Aug 13;30(10):1888–95. doi: 10.1111/sms.13755 (PMC7540343; doi:10.1111/sms.13755)
Supplement: Supplementary file 1 — Supplementary Material [file SMS-30-1888-s001.docx]

**Supplementary material**

**Table 1. Detected and reported use of medications by recreational runners**

| **#** | **Distance** | **NSAIDs/analgesics detected in urine** | **Reported use of**  **medications** | **Specific medications*** |
| --- | --- | --- | --- | --- |
| 1 | **21.1 km** | ibuprofen | Yes | Fenproucomon, tambocor, psyllium supplement, movicolon |
| 2 |  | ibuprofen | No | - |
| 3 |  | paracetamol | Yes | Acetylsalicylic acid, diltiazem |
| 4 |  | ibuprofen | Yes | Allopurinol, quinapril |
| 5 |  | paracetamol | Yes | Acetylsalicylic acid, metoprolol |
| 6 |  | paracetamol | Yes | Losartan, citalopram, omega 3-6-9, multivitamins |
| 7 |  | paracetamol | No | - |
| 8 |  | paracetamol | No | - |
| 9 |  | paracetamol | No | - |
| 10 |  | paracetamol | Yes | Rizatriptan, paracetamol |
| 11 |  | ibuprofen | No | - |
| 12 |  | paracetamol + ibuprofen | No | - |
| 13 |  | paracetamol | Yes | Insulin, ezetrol, metformin, irbesartan, magnesium, vitamin D |
| 14 | **10 km** | ibuprofen | No | **-** |
| 15 |  | naproxen | No | **-** |
| 16 |  | ibuprofen | No | **-** |
| 17 |  | ibuprofen | No | **-** |
| 18 |  | naproxen | No | **-** |

* Participants were asked about the use of medications over one month prior to the race.
